# Increase of Plasma IL-12/p40 Ratio Induced by the Combined Therapy of DNA Vaccine and Lamivudine Correlates with Sustained Viremia Control in CHB Carriers

**DOI:** 10.4110/in.2009.9.1.20

**Published:** 2009-02-28

**Authors:** Se Jin Im, Se-Hwan Yang, Seung Kew Yoon, Young Chul Sung

**Affiliations:** 1 Division of Molecular and Life Sciences, Pohang University of Science & Technology, Pohang, Korea.; 2 Research Institute, Genexine Co. Ltd., Pohang, Korea.; 3 Department of Internal Medicine, College of Medicine, The Catholic University of Korea, Seoul, Korea.

**Keywords:** hepatitis B virus, DNA vaccine, interleukin-12, IL-12/p40 ratio, sustained viremia control

## Abstract

**Background:**

We previously reported that IFN-γ producing T cell responses induced by the combined therapy of DNA vaccine and lamivudine for one year are important for the induction of sustained virological response (SVR). However, IFN-γ production is not sufficient to predict sustained viremia control in chronic hepatitis B (CHB) carriers treated.

**Methods:**

Twelve CHB carriers were intramuscularly immunized 12 times at a 4-week interval with 8 mg of HBV DNA vaccine during the standard lamivudine treatment (100 mg/daily/1 year). The level of cytokines during and after the combined therapy in plasma of all 12 CHB carriers treated was determined by each ELISA kit. Six out of 12 CHB carriers revisited the clinic, and their HBV DNA levels were examined.

**Results:**

The combined therapy increased plasma IL-12 and IL-12/p40 ratio during the treatment (baseline vs. peak level: 41.8±8.3 vs. 163.1±29.2 pg/ml; p<0.01 and 0.96±0.25 vs. 3.58±0.86; p<0.01, espectively), and the peak level of plasma IL-12 and IL-12/p40 ratio was evoked at 6 to 10 months during the combined therapy. In particular, CHB carriers with SVR had two and three-fold higher level of the peak plasma IL-12 and plasma IL-12/p40 ratio than non-virological responders (NVRs), respectively (218.0±41.4 vs. 108.1±28.6 pg/ml; p=0.09 and 5.35±1.38 vs. 1.80±0.29; p<0.05, respectively), while p40 level was consistent during the combined therapy. In addition, there was no significant temporal correlation between the peak IL-12/p40 ratio and the elevation of serum alanine aminotransferase (ALT) in this study, contrast to IFN-α therapy which induced peak IL-12 level following ALT flares.

**Conclusion:**

Our results indicate that the combined therapy induces the increase of plasma IL-12 and IL-12/p40 ratio, which are associated with long-term SVR in CHB carriers.

## INTRODUCTION

Two billion people have been infected with the hepatitis B virus (HBV) all over the world, and 400 million of them are estimated to be chronically infected (1). Despite effective prophylactic vaccines, 50 million people are infected with HBV every year. About 80% of hepatocellular carcinoma (HCC) is caused by persistent chronic hepatitis B (CHB) infection. In addition, CHB carriers are highly susceptible to death by hepatic cirrhosis and HCC (2).

We previously demonstrated that combined therapy of lamivudine and DNA vaccine encoding all of the HBV antigens and a genetically engineered human IL-12 mutant, hIL-12 N222L, induced a sustained virological response (SVR) for at least 1 year after the cessation of the combined therapy in six out of 12 CHB carriers, who are designated virological responders (VRs) (3). The other six carriers, the nonvirological responders (NVRs), had a high level of HBV DNA at the end of the combined therapy and during follow-up periods. In addition, HBeAg seroconversion was observed during the treatment in all 3 HBeAg-positive carriers with sustained virological responses. One of them has shown HBsAg seroconversion at a follow-up period, which is hardly achievable by lamivudine monotherapy (4). Correspondingly, VRs showed significantly stronger HBV-specific Interferon(IFN)-γ responses, mainly CD4^+^ memory T cell responses, than NVRs, both at the end of treatment and during follow-up periods. However, these HBV specific T cell responses are not statistically sufficient for predicting SVR, due to the small number of CHB carriers recruited in this study.

It is well documented that cytokines, such as IFN-α/β, IFN-γ, tumor necrosis factor(TNF)-α, Interleukin(IL)-2, and IL-12, trigger viral clearance in many viral infections (5). In the case of HBV infection, the elimination of HBV was generally mediated by several cytokines such as IFN-α/β and IFN-γ via non-cytolytic mechanism (6). These cytokines contribute to antiviral responses via the degradation of viral RNA by the increase of iNOS (7). In addition, the proteolytic cleavage of La autoantigen induced by theses cytokines also involves in the viral clearance by making viral RNA susceptible to host RNase (8). The previous report demonstrated that serum IL-12 generated by IFN-α therapy is responsible for the viremia control in HBeAg-positive CHB carriers (9), indicating that the cytokines like IL-12 could have a significant role for the suppression of viral replication and would be an indicator to predict the sustained virological responses in CHB carriers.

In order to investigate a correlation between SVR and the level of cytokines induced by the combined therapy with DNA vaccine plus lamivudine, we analyzed the level of several plasma cytokines by ELISA assay. Here, we demonstrated that significant increase of plasma IL-12 and IL-12/p40 ratio was transiently induced during the combined therapy. In addition, SVRs showed higher peak plasma IL-12/p40 ratio than NVRs, indicating a potential biomarker to predict the sustained viremia control in the CHB carriers receiving chemotherapy and antigen-specific immunotherapy.

## MATERIALS AND METHODS

### Patients

**Figure 1:**
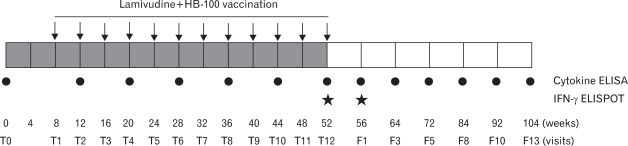
Experimental schedule of the pilot-clinical study. Lamivudine treatment for 8 weeks had been preceded before the first HB-100 injection at T1 except for three subjects; V#118 at week 4, and V#105 and V#106 at week 12. The vaccinees were intramuscularly administrated 12 times with 8 mg of HB-100 every 4 weeks as indicated by the arrows in combination with 100 mg of LAM treatment daily for 52 weeks (gray rectangles) and another 52 weeks were followed (white rectangles). Plasma samples were obtained every 4 weeks (black circles) and the level of cytokines was evaluated by ELISA assay.

We assessed plasma samples from 12 CHB carriers treated with combined therapy of DNA vaccine plus lamivudine as previously reported (3). Briefly, represented in Fig. 1, 12 Caucasian CHB carriers were enrolled from Ukraine and Lithuania and injected 12 times with 8 mg of HB-100 expressing HBV S, pre S1/S2, core, polymerase, X protein and human IL-12 mutant (hIL-12N222L) intramuscularly at four-week interval in combination with 100 mg of lamivudine (Epivir-HBV, GlaxoSmithKline) daily for 52 weeks. Every 4 week, plasma samples were prepared and delivered to a central laboratory at Pohang University of Science and Technology in Korea using a portable liquid nitrogen tank and subsequently used for ELISA assay. The present study was approved by the Pharmacological Center of the Ministry of Health of Ukraine, by the Commission of Non-clinical and Clinical Trials of the State Medicines Control Agency of the Ministry of Health of Lithuania.

Six (V#103, V#106 and V#113; VRs and V#105, V#107 and V#114; NVRs) out of 12 subjects revisited the clinic three years after the cessation of the combined therapy to investigate their long-term virological responses.

### Cytokine ELISA

Plasma samples were diluted 1:2 or 1:5 and then used to measure the levels of cytokines in triplicate. The levels of plasma IL-12, IL-2, IFN-γ, and IL-4 were determined with specific ELISA kits according to the manufacturer's protocols (IL-12, IL-2, and IL-4 : detection limit of 4 pg/ml, IFN-γ: detection limit of 1 pg/ml, BD Biosciences). The levels of plasma p40 subunit were measured using a specific ELISA kit with 2.7% cross-reactivity with recombinant IL-12 protein (detection limit of 32.1 pg/ml, R&D systems).

### Clinical laboratory tests

HBeAg, HBsAg, anti-HBe, and anti-HBs antibodies were measured with commercial enzyme immunoassay (EIA) kits (Monolisa, Biorad). Serum HBV DNA was measured using the Amplicor HBV monitor and Cobas Amplicor HBV monitor test (detection limits of 1,000 and 200 copies/ml, respectively, Roche Diagnostic System), and then data were represented as average values.

### Statistical analysis

Nonparametric tests such as the Wilcoxon signed rank test and Mann-Whitney U test were used for statistical analysis by applying SAS 8.2 for Windows (SAS Institute, Cary, North Carolina, USA) because the population of subjects was relatively small and data did not meet parametric assumptions. The Wilcoxon signed rank test was used to evaluate alterations in levels of IL-12 and the IL-12/p40 ratio in each subject after combined therapy. Comparison of peak IL-12 levels, peak p40 levels and peak IL-12/p40 ratios between virological responders (VRs) and nonvirological responders (NVRs) was determined by the Mann-Whitney U test.

## RESULTS

### Combined therapy of DNA vaccine plus lamivudine led to IL-12 induction during the treatment

**Figure 2:**
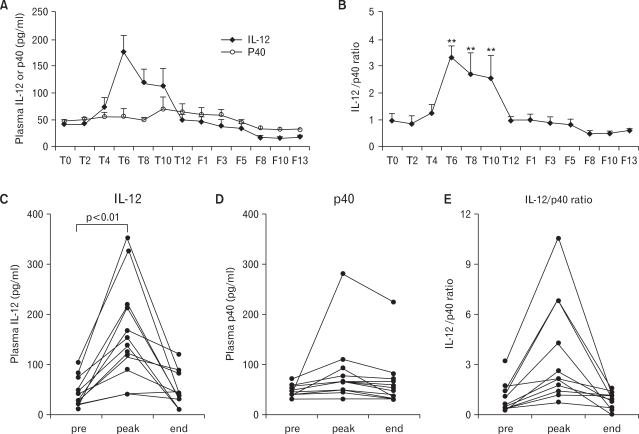
(A, B) Serial analysis of the average level of plasma IL-12, p40 (A), and IL-12/p40 ratio (B) (mean±SEM) in CHB carriers during and after the combined therapy of DNA vaccine plus lamivudine. The level of plasma IL-12 and p40 was determined by a specific ELISA. (^*^p<0.05, ^**^p<0.01 by Wilcoxon signed rank test) (C-E) The level of plasma IL-12 (C), p40 (D), and IL-12/p40 ratio (E) in each subject was shown before the treatment (pre, T0), the peak level detected during the combined therapy (peak, usually T6 to T10), and at the end of treatment (end, T12).

To investigate alterations of immunological characteristics as indicators to predict SVR by the combined therapy, the level of plasma IL-12, p40, IL-4, IL-2, and IFN-γ was monitored longitudinally in each individual by a specific ELISA assay. Among cytokines analyzed, only IL-12 and p40 were detectable. The highest level of plasma IL-12 (163.1±29.2 pg/ml; mean±SEM) was observed 6 to 10 months after combined therapy and was 4-fold higher than pretreatment level on the average (baseline vs. peak level; 41.8±8.3 pg/ml vs. 163.1±29.2 pg/ml; mean±SEM; p<0.01, Wilcoxon signed rank test) (Fig. 2A, C). However, it tended to return to basal levels thereafter. On the other hand, p40, which is known as a natural antagonist against IL-12, was not significantly changed regardless of the treatment except one patient (V#112) (Fig. 2A, D). Similar to plasma IL-12, elevation of the IL-12/p40 ratio (3.58±0.86 from 0.96±0.25, mean±SEM) was detected 6 to 10 months after combined therapy (p<0.01, Wilcoxon signed rank test) (Fig. 2B, E). In an independent experiment, no significant change in plasma IL-12 and IL-12/p40 ratio appeared in any of the 5 CHB carriers treated with lamivudine alone (data not shown), indicating that the increase of plasma IL-12 and IL-12/p40 ratio appears to be associated with DNA vaccine in combination with lamivudine treatment.

### VRs have significantly higher peak IL-12/p40 ratio than NVRs

**Figure 3:**
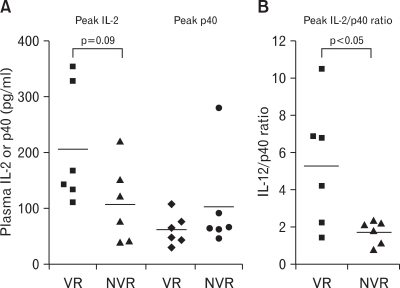
The level of peak plasma IL-12, p40 (A), and peak IL-12/p40 ratio (B) between virological responders (VRs) and nonvirological responders (NVRs) after combined therapy. Peak plasma IL-12 and peak IL-12/p40 were mostly detected from 6 (T6) to 10 (T10) months in 12 CHB carriers treated with combined therapy. The statistical analysis was done by Mann-Whitney U test.

When the peak level of IL-12 was compared between VRs and NVRs, VRs showed approximately a 2-fold higher level of peak plasma IL-12 than NVRs, albeit this was marginally significant (218.0±41.4 vs. 108.1±28.6 pg/ml, respectively; AVR±SEM; p=0.09, Mann-Whitney U) (Fig. 3A). Interestingly, VRs exhibited a significantly higher IL-12/p40 ratio than NVRs (5.35±1.38 vs. 1.80±0.29, respectively; AVR±SEM, p<0.05 Mann-Whitney U) (Fig. 3B). It is worth noting that there is no significant difference in pretreatment levels of plasma IL-12 and IL-12/p40 ratios between VRs and NVRs. Our result is somehow similar to the previous reports that murine recombinant IL-12 (rIL-12) can efficiently suppress HBV replication in transgenic mice (10) and that the recovery from CHB by IFN-α treatment depends on the level of serum IL-12 (9). More importantly, human rIL-12 protein administered subcutaneously inhibits viral replication in CHB patients with a peak serum level of IL-12 (173 pg/ml), which is similar to the peak IL-12 level (163.1 pg/ml) induced in this study (11).

**Figure 4:**
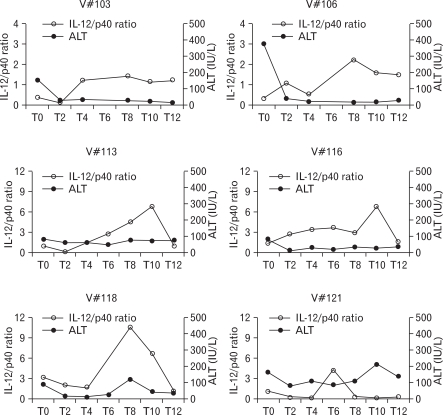
The longitudinal analysis of the level of serum ATL and IL-12/p40 ratio in virological responders during the combined therapy. The kinetics of serum ALT levels (closed circles) and IL-12/p40 ratio (open circles) in VRs was compared in parallel to examine the temporal correlation between them.

In contrast to IFN-α therapy which induced hepatitis flares in 8 out of 10 VRs, followed by peak IL-12 level (9), the elevation of alanine aminotransferase (ALT) level was generated in 2 out of 6 VRs during the treatment, and only one patient (V#118) showed concomitant elevation of ALT level with the increased IL-12/p40 ratio (Fig. 4), reflecting that there was no significant temporal correlation between the increase of plasma IL-12 and an ALT flare in this study. These results indicate that our combined therapy could suppress viral replications presumably by the increased IL-12 without massive hepatocytolysis.

### Virological responses are maintained in VRs for 3 years after stopping combined therapy

**Figure 5:**
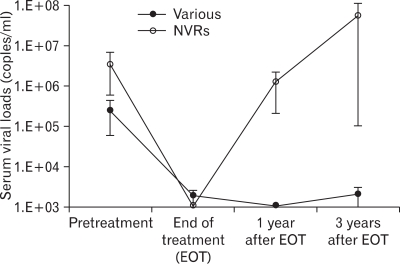
The longitudinal analysis of serum viral loads for up to 3 years in CHB carriers treated with combined therapy. Three VRs (V#103, V#106 and V#113; closed circles) and 3 NVRs (V#105, V#107 and #114; open circles) out of 12 subjects revisited the clinic 3 years after the stopping the combined therapy. Data were represented by the average serum viral loads according to the time point. (mean±SEM).

Previously, we demonstrated that there were 6 VRs and 6 NVRs, as determined by viral loads at the end of a 1-year follow-up. Among them, six subjects (V#103, V#106 and V#113; VRs and V#105, V#107 and V#114; NVRs) have revisited the clinic 3 years after the cessation of combined therapy. When their serum viral loads were determined to address long-term efficacy of combined therapy, two (V#103, V#106) out of 3 VRs still showed undetectable serum viremia, and one subject (V#113) had a very low level of serum viral load (3,200 copies/ml), reflecting the maintained viremia control for up to 3 years in VRs after the combined therapy (Fig 5). Interestingly, it is likely that all 3 VRs showed strong HBV antigen-specific IFN-γ responses or higher IL-12/p40 ratio (Table I), suggesting that they might be involved in controlled viremia in vaccinees. As expected, one subject (V#106) still maintained HBsAg seroconversion (data not shown). In contrast, all 3 NVRs had high viral loads 3 years after the cessation of combined therapy, even though two of them (V#105 and V#107) had received IFN-α therapy for 6 months after the follow-up period, and their serum viral loads reached more than 200,000 and 162,000,000 copies/ml, respectively.

## DISCUSSION

This experiment is the first finding to investigate plasma IL-12 and the IL-12/p40 ratio longitudinally after active immunotherapy such as DNA vaccine together with chemotherapy, which correlate with SVR in CHB carriers treated. In addition, we showed that long-term therapeutic efficacy had been sustained for at least 3 years after the cessation of the combined therapy.

Like IFN-α therapy (9), combined therapy of DNA vaccine and lamivudine evoked an increase in peak IL-12 levels and peak IL-12/p40 ratio in CHB carriers during the treatment. Different with IFN-α therapy, however, increase of plasma IL-12 and IL-12/p40 ratio did not correlate directly with the elevation of ALT levels in this study. This discrepancy could be explained as follows: It is possible that major cell types secreting IL-12 are different between IFN-α therapy and our combined therapy. IFN-α treatment leads to systemic immune stimulation and hepatocellular necrosis, recruiting macrophages and noncommitted T cells into the liver (9). In contrast, after the combined therapy, IL-12 could be mainly secreted by activated dendritic cells stimulated by DNA vaccination under lamivudine treatment in the secondary lymphoid organs, not in the liver, resulting in generation of antigen-specific type I T helper responses. Such Th1 responses represented by secretion of IFN-γ and other Th1 cytokines resulted in viral suppression via non-cytolytic mechanisms (12), such as elimination of HBV nucleocapsid particles and destabilization of the viral RNA. In addition, it is likely that ALT flares occur in proportion to the level of serum/plasma IL-12. The concentration of plasma IL-12 detected after our combined therapy reaches several hundred pg/ml. On the other hand, IFN-α therapy induced more than 10 ng/ml of peak serum IL-12, which mediated excessive inflammation and destruction of hepatocytes (9).

It is arguable that the observed plasma IL-12 may be derived from the injected plasmid encoding hIL-12 mutant included in our DNA vaccine or not. However, the plasma IL-12 is likely to be synthesized by the host immune system stimulated by the DNA vaccine on the basis of the following observations: DNA vaccine was administered twelve times at a 4-week interval, but plasma IL-12 was induced only from T4 to T10 in the vaccinees, and disappeared thereafter. In addition, it was previously reported that gene expression of the injected DNA reached its peak level on days 1~3, and then declined to the basal level after 2 weeks in the muscle (13). Similarly, when mice were injected with 100 µg of hIL-12N222L (>100X of human dose as calculated with body weight), low levels of peak human IL-12 (25±3.2 pg/ml, mean±SEM) were observed only in the muscle on the second day after injection, but was not detected in plasma and serum during the month (our unpublished data), possibly excluding the production of plasma IL-12 from injected plasmid itself.

Although both IL-12 and IFN-γ production are considered as Th1 responses, two patients (V#116, V#118) had weak IFN-γ responses even though they showed higher IL-12/p40 ratio. It is likely that the discrepancy between IFN-γ responses and IL-12/p40 ratio relied on the time point analyzed, that is, peak IL-12/p40 ratio was generated at T6 to T10 in most cases but IFN-γ responses were determined at only T12 or F1. To clarify the correlation between IL-12/p40 ratio and IFN-γ responses, the longitudinal analysis of IFN-γ responses during the treatment should be performed for a next clinical study.

Because there was no significant correlation between IL-12 induction and IFN-γ responses in each individual, the IL-12 induction could be regarded as an independent parameter to effect on the SVR in CHB carriers treated with the combined therapy (Table I). In addition, according to our previous and present study, one parameter such as IFN-γ production or IL-12/p40 ratio alone is not sufficient to predict SVR in CHB carriers treated although NVRs showed both weak IFN-γ responses and low IL-12/p40 ratio. Combined both parameters, we suggested that "the level of the plasma IL-12/p40 ratio or the number of env-specific IFN-γ secreting cells/10^6^ PBMCs as determined by ex-vivo ELISPOT assay" could be used as an early biomarker alternatively to predict a long-term SVR after stopping the combined therapy. All NVRs had fewer than 200 env-specific IFN-γ secreting cells per 10^6^ PBMCs and less than 3 of peak IL-12/p40 ratio (0.79~2.67, 1.8±0.3; mean±SEM). In contrast, all VRs showed more than 300 env-specific IFN-γ secreting cells per 10^6^ PBMCs or more than 4 of peak IL-12/p40 ratio (Table I). Taken together, it is possible to suggest that vaccinees showing>4 peak IL-12/p40 ratio or >300 env-specific IFN-γ secreting cells/10^6^ PBMCs by *ex vivo* ELISPOT assay could suppress HBV replication for a long time after stopping the combined therapy, indicating the possibility as novel early biomarkers. However, since we analyzed the small number of CHB carriers, our observations require further validation in a larger cohort of CHB carriers treated with the combined therapy.

In conclusion, our study suggests that combined therapy of DNA vaccine and lamivudine leads to the induction of plasma IL-12 and IL-12/p40 ratio. In addition, the elevation of plasma IL-12/p40 ratio as well as HBV-specific IFN-γ responses is closely associated with the viremia control in CHB carriers, and could be used for early biomarkers to predict the SVR after the cessation of the combined therapy.

## Figures and Tables

**Table I:**
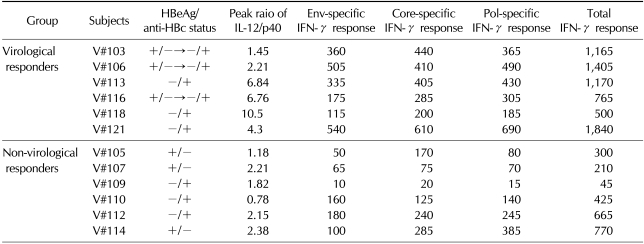
The presence of HBeAg/anti-HBe, peak IL-12/p40 ratio, and HBV-specific T cell responses determined by *ex vivo* ELISPOT in each CHB carrier

HBV antigen-specific IFN-γ response reflects the number of spots per 10^6^ PBMCs determined by IFN-γ ELISPOT assay excluding that generated by HCV E2 peptide.

## References

[1] Lok AS, Heathcote EJ, Hoofnagle JH (2001). Management of hepatitis B: 2000--summary of a workshop. Gastroenterology.

[2] de Jongh FE, Janssen HL, de Man RA, Hop WC, Schalm SW, van Blankenstein M (1992). Survival and prognostic indicators in hepatitis B surface antigen-positive cirrhosis of the liver. Gastroenterology.

[3] Yang SH, Lee CG, Park SH, Im SJ, Kim YM, Son JM, Wang JS, Yoon SK, Song MK, Ambrozaitis A, Kharchenko N, Yun YD, Kim CM, Kim CY, Lee SH, Kim BM, Kim WB, Sung YC (2006). Correlation of antiviral T-cell responses with suppression of viral rebound in chronic hepatitis B carriers: a proof-of-concept study. Gene Ther.

[4] Lai CL, Chien RN, Leung NW, Chang TT, Guan R, Tai DI, Ng KY, Wu PC, Dent JC, Barber J, Stephenson SL, Gray DF, Asia Hepatitis Lamivudine Study Group (1998). A one-year trial of lamivudine for chronic hepatitis B. N Engl J Med.

[5] Guidotti LG, Chisari FV (2000). Cytokine-mediated control of viral infections. Virology.

[6] Chisari FV, Ferrari C (1995). Hepatitis B virus immunopathogenesis. Annu Rev Immunol.

[7] Guidotti LG, McClary H, Loudis JM, Chisari FV (2000). Nitric oxide inhibits hepatitis B virus replication in the livers of transgenic mice. J Exp Med.

[8] Heise T, Guidotti LG, Chisari FV (2001). Characterization of nuclear RNases that cleave hepatitis B virus RNA near the La protein binding site. J Virol.

[9] Rossol S, Marinos G, Carucci P, Singer MV, Williams R, Naoumov NV (1997). Interleukin-12 induction of Th1 cytokines is important for viral clearance in chronic hepatitis B. J Clin Invest.

[10] Cavanaugh VJ, Guidotti LG, Chisari FV (1997). Interleukin-12 inhibits hepatitis B virus replication in transgenic mice. J Virol.

[11] Carreño V, Zeuzem S, Hopf U, Marcellin P, Cooksley WG, Fevery J, Diago M, Reddy R, Peters M, Rittweger K, Rakhit A, Pardo M (2000). A phase I/II study of recombinant human interleukin-12 in patients with chronic hepatitis B. J Hepatol.

[12] Guidotti LG, Ishikawa T, Hobbs MV, Matzke B, Schreiber R, Chisari FV (1996). Intracellular inactivation of the hepatitis B virus by cytotoxic T lymphocytes. Immunity.

[13] Barouch DH, Truitt DM, Letvin NL (2004). Expression kinetics of the interleukin-2/immunoglobulin (IL-2/Ig) plasmid cytokine adjuvant. Vaccine.

